# The Contribution of Alexithymia to Obsessive-Compulsive Disorder Symptoms Dimensions: An Investigation in a Large Community Sample in Italy

**DOI:** 10.1155/2015/707850

**Published:** 2015-09-06

**Authors:** Andrea Pozza, Nicoletta Giaquinta, Davide Dèttore

**Affiliations:** ^1^Department of Experimental and Clinical Medicine, University of Florence, Largo Brambilla 3, 53100 Florence, Italy; ^2^Miller Institute of Behavioral and Cognitive Psychotherapy, Corso Torino 19/2, 16129 Genoa, Italy; ^3^Department of Health Sciences, University of Florence, Via di San Salvi 12, Building 26, 50135 Florence, Italy

## Abstract

Poor attention has been dedicated to the relation between Alexithymia and specific OCD symptoms dimensions. Knowledge about which Alexithymia domains are the most affected ones in OCD dimensions could inform clinical practice, suggesting the need for the introduction of psychotherapeutic interventions targeting Alexithymia deficits. The current study aimed to investigate which OCD symptom dimension correlated with Alexithymia domains. A total of 425 community individuals (mean age = 27.80, SD = 9.89, 60% women) completed measures of Alexithymia, OCD symptoms dimensions, anxiety, and depression. Moderate correlations emerged between Difficulty Identifying Feelings and Hoarding (*r* = .36, *p* < .001) and Checking symptoms (*r* = .34, *p* < .001) and between Difficulty Describing Feelings and Pure Obsessing (*r* = .31, *p* < .001). Difficulty Identifying Feelings uniquely predicted OCD symptoms (*β* = 0.20, *t* = 3.96, and *p* < .001), after controlling for anxiety and depression. A main effect emerged of Alexithymia on Ordering (*β* = 0.70, *t* = 2.50, *p* < .05) and Pure Obsessing symptoms (*β* = 0.043, *t* = 2.08, and *p* < .05). Psychotherapeutic interventions specifically targeting Alexithymia should be integrated in the treatment of Ordering and Pure Obsessing symptoms. Difficulty Identifying Feelings and Difficulty Describing Feelings should be addressed in the psychotherapeutic treatment of Hoarding, Checking, and Pure Obsessing, respectively.

## 1. Introduction

Obsessive-Compulsive Disorder (OCD) is a chronic psychological condition with a lifetime prevalence of 2% in the general population [1, 2]. OCD consists of intrusive thoughts, impulses, or mental images and repetitive behaviours or mental compulsions, which can strongly affect quality of life of the individual [3]. OCD symptoms are phenomenologically heterogeneous and etiologically complex [3]. The World Health Organization has ranked OCD as the tenth leading cause of disability of all health conditions in the industrialized world [3]. Studies of analogue samples (i.e., student and community participants) highlighted the prevalence of subclinical OCD symptoms [1–3]. According to surveys, up to 90% of people report that they occasionally experience intrusive thoughts, which are similar in form and content to clinical obsessions [3].

The construct of Alexithymia indicates a cluster of cognitive and affective characteristics, including difficulties in recognizing and verbalizing feelings, paucity of fantasy life, concrete speech, and thought closely tied to external events [4]. Some evidence suggested that alexithymic characteristics seem to be a temporally stable trait in OCD [4–7]. Bankier et al. [8] compared alexithymic characteristics in a sample of 234 patients with different psychiatric disorders, including somatoform disorder, panic disorder, OCD, and depression. Findings showed that in contrast to those with panic disorder patients with OCD tended to cope with emotional stress by the use of an operational thinking style [8].

Kang and colleagues [9] reported that patients with OCD had lower levels of perspective taking and higher levels of Alexithymia relative to sex-matched healthy controls. In addition, patients with OCD had lower empathic ability in perspective taking and a perception bias towards disgust in response to ambiguous facial expressions [9].

Recently, Robinson and Freeston [10] summarized evidence through a systematic review of Alexithymia in OCD. By a hand search of electronic databases (Medline, Embase, PsycInfo, Web of Knowledge, and Scopus), the authors identified five studies, which indicated that patients with OCD had significantly higher levels on all the three Alexithymia domains, although in one study differences between the two groups were not significant on Externally Oriented Thinking. Only one study [11] explored the relationship between OCD symptom dimensions and Alexithymia through a behavior-based measure of OCD. Regression analysis indicated that the sexual/religious dimension [11] was the only symptom predictor of the TAS-20 Total scores, whereas the other four symptom dimensions in this study (symmetry/Ordering, Hoarding, contamination/cleaning, and aggressive/Checking) did not show a significant relationship with the TAS total scores.

In conclusion, poor attention has been dedicated to the relation between Alexithymia and specific OCD symptoms dimensions. Knowledge about which Alexithymia domains are most affected in specific OCD symptoms dimensions could inform clinical practice suggesting the need for the introduction of therapeutic components targeting specific Alexithymia deficits in the treatment of specific OCD dimensions.

Therefore, the current study aimed to investigate which OCD symptom dimensions are uniquely predicted by Alexithymia domains in a large community sample after controlling for anxiety and depression.

## 2. Materials and Methods

### 2.1. Participants

A total of 425 community individuals participated in the study (60% women). Mean age was 27.80 years (SD = 9.89, range = 18–76). All participants were white. Marital status was 86% single, 10% married or cohabitating, 3.10% separated or divorced, and 0.50% widowed. Forty-five percent of the sample was students, 23% had a full- or part-time job, and 6% was unemployed or retired. Participants were unscreened in order to obtain a more representative sample of the community population.

Data collection was carried out from November 2013 to July 2014. Through convenience sampling, participants were recruited in a variety of public settings in several cities located in the Northern, Mid, or Southern Italy. Psychologists approached participants in public settings, including high schools, universities, railway stations, libraries, malls, sports, or volunteering associations. When approached, each participant was provided with a brief overview of the study. If interested, he/she was taken aside to complete the questionnaires individually or in small groups. In accordance with the Ethical Principles of Psychologists and Code of Conduct [12], all the participants who were recruited provided written informed consent to participate after having received a detailed description of the study aims. Participants' identities remained anonymous and participation was entirely volunteer and uncompensated. Contact information of the study coordinator (DD) was provided if participants had further questions or concerns regarding their participation.

### 2.2. Measures

Participants completed a questionnaire on sociodemographic information and a packet of self-report clinical scales, including the Beck Anxiety Inventory (BAI; [13]), the Beck Depression Inventory-II (BDI-II; [14]), the Obsessive-Compulsive Inventory-Revised (OCI-R; [15]), and the Toronto Alexithymia Scale-20 (TAS-20; [16]).

The BAI [13] was used to measure anxiety symptoms. It is a self-report questionnaire consisting of 21 items. The Italian version showed good internal consistency (Cronbach's alpha = 0.87) [17]. In the current study internal consistency was excellent (Cronbach's alpha = 0.90).

The BDI-II [14] was used to assess depressive symptoms. It is a 21-item self-reporting inventory rating the severity of depressive symptoms. Items are rated from 0 to 3, and the total score ranges from 0 to 63. Higher scores denote higher levels of depression. The Italian version [18] has shown excellent internal consistency (Cronbach's alpha = .93). In the current study alpha was excellent (alpha = .90).

The OCI-R [15] was used to assess OCD symptoms subtypes. It is a self-report measure consisting of 18 items, which assess six OCD symptom subtypes:* Washing*,* Obsessing*,* Hoarding*,* Ordering*,* Checking*, and* Mental Neutralizing*. The Italian version [19] showed good internal consistency for all the six subscales (0.76 < Cronbach's alpha < 0.94), except for the Washing subscale (Cronbach's alpha = 0.60) and Mental Neutralizing (Cronbach's alpha = 0.61). In the current study internal consistency was very good (Cronbach's alpha = 0.88).

The TAS-20 [16], the most widely used measure of Alexithymia, has a three-factor structure, consisting of Difficulty Identifying Feelings (the capacity to identify feelings and to distinguish between feelings and the bodily sensations of emotional arousal), Difficulty Describing Feelings (the inability to communicate feelings to other people), and Externally Oriented Thinking (i.e., paucity of fantasy life, concrete speech, and thought closely tied to external events). The Italian TAS-20 [20] showed good internal consistency (Cronbach's alpha = 0.81). In the current study internal consistency was very good (Cronbach's alpha = 0.84).

An overview of mean scores and standard deviations of the sample (*n* = 425) on all the clinical scales is presented in Table 1.

### 2.3. Statistical Analysis

To investigate the relations between Alexithymia dimensions and OCD symptoms dimensions, bivariate correlations were performed computing Pearson's *r* coefficients between TAS-20 and OCI-R subscales scores. Power calculations were run for this analysis. For a medium effect size, 80% power, and significance set at *p* < .001, the required sample size for bivariate correlations was 162.

To test the unique contribution of Alexithymia to OCD symptoms after controlling for depression and anxiety, stepwise linear regression models were performed entering BDI-II, BAI, and TAS-20 subscale scores as predictors and OCI-R Total scores as outcomes. Power calculations were run for this analysis. For a medium effect size, 80% power, and significance set at *p* < .001, the required sample size for bivariate correlations was 152.

To examine the effects of Alexithymia dimensions on OCD symptoms dimensions, multiple linear regression models were performed entering BDI-II, BAI, and TAS-20 subscale scores as independent variables and OCI-R subscale scores as outcomes. Between-group effect sizes were estimated using the partial eta squared index as recommended by Olejnik and Algina [21]. According to Cohen [22], effect sizes of 0.01, 0.06, and 0.14 were interpreted as small, medium, and large, respectively.

Statistical analysis was conducted with SPSS software version 21.00.

## 3. Results

### 3.1. Relations between Alexithymia Domains and OCD Symptoms Dimensions

Significant moderate correlations emerged between scores on the TAS-20 Total and the OCI-R Total (*r* = .44, *p* < .001), the OCI-R Hoarding (*r* = .33, *p* < .001), and OCI-R Total scores (*r* = .43, *p* < .001). Significant low correlations emerged between scores on the TAS-20 Total and the OCI-R Checking (*r* = .29, *p* < .001), the OCI-R Ordering (*r* = .30, *p* < .001), the OCI-R Neutralizing (*r* = .17, *p* < .001), and the OCI-R Washing scores (*r* = .23, *p* < .001).

Significant moderate correlations emerged between scores on the TAS-20 Difficulty Identifying Feelings (TAS-20 DIF), scores on the OCI-R Total (*r* = .49, *p* < .001), the OCI-R Hoarding (*r* = .36, *p* < .001), and the OCI-R Checking (*r* = .34, *p* < .001), and low correlations emerged between scores on the TAS-20 DIF, OCI-R Ordering (*r* = .29, *p* < .001), OCI-R Neutralizing (*r* = .18, *p* < .001), OCI-R Washing (*r* = .25, *p* < .001), and the OCI-R Obsessing (*r* = .56, *p* < .001).

Significant moderate correlations emerged between scores on the TAS-20 Difficulty Describing Feelings (TAS-20 DDF), scores on the OCI-R Total (*r* = .32, *p* < .001), and OCI-R Obsessing (*r* = .31, *p* < .001), and low correlations emerged between the TAS-20 DDF, scores on the OCI-R Hoarding (*r* = .26, *p* < .001), OCI-R Checking (*r* = .20, *p* < .001), Ordering (*r* = .24, *p* < .001), and OCI-R Washing (*r* = .18, *p* < .001), and nonsignificant correlations emerged between TAS-20 and OCI-R Neutralizing. Bivariate correlations between the TAS-20 and OCI-R subscales are provided in Table 2.

### 3.2. Alexithymia as Unique Predictor of OCD Symptoms Dimensions

Linear regression analyses were conducted to test whether Alexithymia domains predicted OCD symptoms after controlling for anxiety and depression. Results showed that scores on the TAS-20 Difficulty Identifying Feelings significantly and uniquely predicted OCI-R Total scores (*β* = 0.20, *t* = 3.86, *p* < .001, and *R* ^2^ = 0.03), after controlling for the effects of BDI-II (*β* = 0.24, *t* = 4.19, *p* < .001, and *R* ^2^ = 0.28) and BAI scores (*β* = 0.26, *t* = 4.84, *p* < .001, and *R* ^2^ = 0.06). The linear regression model built entering BDI-II scores, BAI scores, all the three TAS-20 subscale scores, and OCI-R scores as outcomes explained 37% of total variance. Beta coefficients of BDI-II, BAI, and TAS-20 scores on the OCI-R Total scores are provided in Table 3.

Multiple linear regression analyses were conducted to test the contribution of BAI, BDI-II, and TAS-20 scores on the OCI-R subscales. Results indicated only a main effect of BDI-II scores on the OCI-R Obsessing scores (*β* = 0.23, *t* = 2.69, *η* ^2^ = 0.02, and *p* < .05), a main effect of TAS-20 scores on the OCI-R Ordering scores (*β* = 0.70, *t* = 2.50, *η* ^2^ = 0.01, and *p* < .05) and the OCI-R Obsessing (*β* = 0.043, *t* = 2.08, *η* ^2^ = 0.01, and *p* < .05). An overview of results of multiple linear regression analyses with main and interaction effects of BDI-II, BAI, and TAS-20 scores on the OCI-R subscale scores is provided in Table 4.

## 4. Discussion

Poor attention has been dedicated to the relation between Alexithymia domains and OCD. The current findings extended previous data, indicating that specific alexithymic characteristics may be implicated in specific OCD symptoms dimensions.

The present findings suggested that global Alexithymia may be specifically associated only with Ordering and Pure Obsessing OCD symptoms but not with the other OCD dimensions. In addition, moderate correlations emerged between Difficulty Identifying Feelings, Hoarding, and Checking symptoms. Overall, these findings could suggest that psychotherapeutic interventions for Hoarding and Checking dimensions should target Difficulty Identifying Feelings and interventions for individuals with Ordering and Pure Obsessions should include modules dedicated to emotional awareness.

These data could be explained by the fact that individuals with Hoarding and Checking symptoms tend to use compulsive behaviours as a coping strategy for negative emotions due to their strong intolerance for negative feelings. An alternative explanation could be that individuals with Hoarding symptoms, who often have excessive emotional attachment to inanimate objects, have impaired emotional awareness and mental representations about the self and others [23, 24]. In addition, Difficulty Identifying Feelings could explain why patients with Hoarding have generally a poorer response to cognitive behavioural therapy relative to nonhoarder patients with OCD, since cognitive behavioural therapy for OCD traditionally does not focus on emotional awareness [7, 25]. In addition, Difficulty Identifying Feelings may be associated with a poorer insight of symptoms, and this aspect could explain why individuals with Hoarding have a negative response to treatment, since unawareness of anxiety and negative feelings related to intrusions could impede construction of hierarchy or progress of exposure. Poorer emotional awareness could also impede the patient to confront anxiety-evoking intrusive thoughts. Another finding was that OCD symptom dimensions were not associated with Externally Oriented Thinking, suggesting that individuals with OCD symptoms do not have paucity of fantasy life, concrete speech, and thought closely tied to external events. This data was consistent with previous indications [10], confirming that individuals with OCD symptoms do not have deficits in introspective abilities.

Finally, some limitations should be noted. Although some authors believed that OCD symptoms are dimensional rather than categorical in their frequency and severity distributions and that community samples are relevant for investigating OCD phenomena [26], it should be noted that the current study did not use a clinical sample. Thus, future studies should use patients presenting with specific OCD symptoms dimensions. Moreover, the current study did not use a longitudinal design. Therefore, further research should prospectively examine the causal role of Alexithymia as a vulnerability factor implicated in the development of OCD symptoms. Finally, another limitation concerns the use of self-report measures. Although the TAS-20 is believed to be the best validated tool to assess Alexithymia, it has been suggested that studies should be conducted through a multimethod approach for the assessment of Alexithymia, including also non-self-report measures [27], such as the Levels of Emotional Awareness Scale [28]. In effect, it could be argued that individuals with alexithymic characteristics, who are characterized by a diminished affective insight, could not give an accurate estimation of their affective disturbances [27]. Consistent with this hypothesis, Waller and Scheidt [29] reported that patients with Somatoform Disorders had higher scores on the TAS-20 compared to healthy controls but not on non-self-report measures. In addition, only the cognitive domain of the Alexithymia construct, Externally Oriented Thinking, was related to non-self-report measures [29]. Overall, these previous findings, which should be tested also with OCD patients, could suggest that future studies should consider also the inclusion of non-self-report measures to investigate the role of Alexithymia in OCD symptom dimensions.

## 5. Conclusions

In conclusion, the current study extended previous knowledge indicating that alexithymic characteristics related to Difficulty Identifying Feelings seem to be associated with OCD, Hoarding, and Checking symptoms, specifically. Therefore, treatment strategies should focus on targeting this alexithymic domain for individuals presenting with these OCD dimensions.

## Figures and Tables

**Table 1:** Means (standard deviations) on the BAI, BDI-II, TAS-20, and OCI-R (*n* = 425).

	M (SD)
BAI	11.76 (8.93)
BDI-II	8.97 (7.94)
TAS-20 Total	43.70 (11.47)
TAS-20 DIF	14.59 (5.54)
TAS-20 DDF	12.50 (4.82)
TAS-20 EOT	16.61 (4.69)
OCI-R Total	13.65 (10.66)
OCI-R Washing	1.45 (2.06)
OCI-R Obsessing	2.80 (2.99)
OCI-R Hoarding	2.79 (2.81)
OCI-R Ordering	3.25 (2.99)
OCI-R Checking	2.45 (2.64)
OCI-R Mental Neutralizing	0.87 (1.84)

Note: BDI-II = Beck Depression Inventory-II, BAI = Beck Anxiety Inventory, OCI-R = Obsessive-Compulsive Inventory-Revised, TAS-20 = Toronto Alexithymia Scale-20. DIF = Difficulty Identifying Feelings, DDF = Difficulty Describing Feelings, EOT = Externally Oriented Thinking.

**Table 2:** Bivariate correlations between OCI-R subscales and TAS-20 (*n* = 425).

	OCI-R Total	OCI-R Hoarding	OCI-R Checking	OCI-R Ordering	OCI-R Neutralizing	OCI-R Washing	OCI-R Obsessing
TAS-20 Total	.44^*∗*^	.33^*∗*^	.29^*∗*^	.30^*∗*^	.17^*∗*^	.23^*∗*^	.43^*∗*^
TAS-20 DIF	.49^*∗*^	.36^*∗*^	.34^*∗*^	.29^*∗*^	.18^*∗*^	.25^*∗*^	.56^*∗*^
TAS-20 DDF	.32^*∗*^	.26^*∗*^	.20^*∗*^	.24^*∗*^	.06	.18^*∗*^	.31^*∗*^
TAS-20 EOT	.16^*∗*^	.10^*∗*^	.11^*∗*^	.16^*∗*^	.13^*∗*^	.09	.07

Note: OCI-R = Obsessive-Compulsive Inventory-Revised, TAS-20 = Toronto Alexithymia Scale-20. DIF = Difficulty Identifying Feelings, DDF = Difficulty Describing Feelings, EOT = Externally Oriented Thinking.

^*∗*^ *p* < .001 (2-tail).

**Table 3:** Beta coefficients of BDI-II, BAI, and TAS-20 DIF on the OCI-R Total (*n* = 425).

	*β*	*t*	*R* ^2^ change
BDI-II	0.24^*∗*^	4.19	0.28
BAI	0.26^*∗*^	4.84	0.34
TAS-20 DIF	0.20^*∗*^	3.86	0.37

Note: BDI-II = Beck Depression Inventory-II, BAI = Beck Anxiety Inventory, OCI-R = Obsessive-Compulsive Inventory-Revised, TAS-20 = Toronto Alexithymia Scale-20 Difficulty Identifying Feelings.

^*∗*^ *p* < .001.

**Table 4:** Main and interaction effects of BDI-II, BAI, and TAS-20 on the OCI-R subscales (*n* = 425).

		*β*	*t*	*η* ^2^
Main effect of BDI-II	OCI-R Hoarding	0.17	1.87	0.01
	OCI-R Checking	0.01	0.13	0.001
	OCI-R Ordering	−0.11	−1.03	0.003
	OCI-R Neutralizing	−0.05	−0.85	0.003
	OCI-R Washing	−0.07	−0.93	0.003
	OCI-R Obsessing	0.23	2.69^*∗*^	0.02

Main effect of BAI	OCI-R Hoarding	−0.08	−1.11	0.003
	OCI-R Checking	0.17	0.24	0.001
	OCI-R Ordering	0.13	1.66	0.001
	OCI-R Neutralizing	0.05	1.00	0.002
	OCI-R Washing	0.08	1.50	0.01
	OCI-R Obsessing	0.03	0.50	0.01

Main effect of TAS-20	OCI-R Hoarding	0.03	1.61	0.01
	OCI-R Checking	0.03	1.53	0.01
	OCI-R Ordering	0.50	2.70^*∗*^	0.01
	OCI-R Neutralizing	−0.01	−1.01	0.003
	OCI-R Washing	0.02	1.40	0.01
	OCI-R Obsessing	0.04	2.08^*∗*^	0.01

BDI-II × BAI interaction effect	OCI-R Hoarding	0.002	1.05	0.003
	OCI-R Checking	0.001	2.30	0.01
	OCI-R Ordering	0.001	0.45	0.001
	OCI-R Neutralizing	−0.02	−1.74	0.01
	OCI-R Washing	0.001	0.02	0.001
	OCI-R Obsessing	0.001	0.24	0.001

BDI-II × TAS-20 interaction effect	OCI-R Hoarding	−0.01	−1.60	0.005
	OCI-R Checking	0.001	−0.24	0.001
	OCI-R Ordering	0.03	1.37	0.001
	OCI-R Neutralizing	0.002	1.78	0.003
	OCI-R Washing	0.01	1.19	0.004
	OCI-R Obsessing	0.002	1.26	0.004

BAI × TAS-20 interaction effect	OCI-R Hoarding	0.003	1.75	0.001
	OCI-R Checking	0.001	0.09	0.001
	OCI-R Ordering	−0.01	−1.28	0.001
	OCI-R Neutralizing	0.001	0.08	0.001
	OCI-R Washing	0.001	1.00	0.001
	OCI-R Obsessing	−0.01	−1.43	0.001

Note: BDI-II = Beck Depression Inventory-II, BAI = Beck Anxiety Inventory, OCI-R = Obsessive-Compulsive Inventory-Revised, TAS-20 = Toronto Alexithymia Scale-20.

^*∗*^ *p* < .05 (2-tail).

## References

[1] Crino R., Slade T., Andrews G. (2005). The changing prevalence and severity of obsessive-compulsive disorder criteria from DSM-III to DSM-IV. *American Journal of Psychiatry*.

[2] Kessler R. C., Berglund P., Demler O., Jin R., Merikangas K. R., Walters E. E. (2005). Lifetime prevalence and age-of-onset distributions of DSM-IV disorders in the national comorbidity survey replication. *Archives of General Psychiatry*.

[3] Murray C., Lopez A. (1996). *The Global Burden of Disease: A Comprehensive Assessment of Mortality and Disability from Diseases, Injury, and Risk Factors in 1990 and Projected to 2020*.

[4] De Berardis D., Campanella D., Gambi F. (2005). Insight and alexithymia in adult outpatients with obsessive-compulsive disorder. *European Archives of Psychiatry and Clinical Neuroscience*.

[5] de Berardis D., Campanella D., Nicola S. (2008). The impact of alexithymia on anxiety disorders: a review of the literature. *Current Psychiatry Reviews*.

[6] Grabe H. J., Ruhrmann S., Ettelt S. (2006). Alexithymia in obsessive-compulsive disorder—results from a family study. *Psychotherapy and Psychosomatics*.

[7] Rufer M., Ziegler A., Alsleben H. (2006). A prospective long-term follow-up study of alexithymia in obsessive-compulsive disorder. *Comprehensive Psychiatry*.

[8] Bankier B., Aigner M., Bach M. (2001). Alexithymia in DSM-IV disorder: comparative evaluation of somatoform disorder, panic disorder, obsessive-compulsive disorder, and depression. *Psychosomatics*.

[9] Kang J. I., Namkoong K., Yoo S. W., Jhung K., Kim S. J. (2012). Abnormalities of emotional awareness and perception in patients with obsessive-compulsive disorder. *Journal of Affective Disorders*.

[10] Robinson L. J., Freeston M. H. (2014). Emotion and internal experience in obsessive compulsive disorder: reviewing the role of alexithymia, anxiety sensitivity and distress tolerance. *Clinical Psychology Review*.

[11] Roh D., Kim W.-J., Kim C.-H. (2011). Alexithymia in obsessive-compulsive disorder: clinical correlates and symptom dimensions. *Journal of Nervous and Mental Disease*.

[12] American Psychological Association (2002). Ethical principles of psychologists and code of conduct. *American Psychologist*.

[13] Beck A. T., Epstein N., Brown G., Steer R. A. (1988). An inventory for measuring clinical anxiety: psychometric properties. *Journal of Consulting and Clinical Psychology*.

[14] Beck A. T., Steer R. A., Brown G. K. (1996). *Beck Depression Inventory*.

[15] Foa E. B., Huppert J. D., Leiberg S. (2002). The obsessive-compulsive inventory: development and validation of a short version. *Psychological Assessment*.

[16] Bagby R. M., Parker J. D. A., Taylor G. J. (1994). The twenty-item Toronto Alexithymia scale—I. Item selection and cross-validation of the factor structure. *Journal of Psychosomatic Research*.

[17] Sica C., Coradeschi D., Ghisi M., Sanavio E., Beck A. T., Steer R. A. (2006). L'adattamento italiano del BAI. *Beck Anxiety Inventory*.

[18] Ghisi M., Flebus G. B., Montano A., Sanavio E., Sica C., Beck A. T., Steer R. A., Brown G. K. (2006). L’adattamento italiano del BDI-II. *Beck Depression Inventory-II*.

[19] Sica C., Ghisi M., Altoè G. (2009). The Italian version of the Obsessive Compulsive Inventory: its psychometric properties on community and clinical samples. *Journal of Anxiety Disorders*.

[20] Bressi C., Taylor G., Parker J. (1996). Cross validation of the factor structure of the 20-item Toronto Alexithymia Scale: an Italian multicenter study. *Journal of Psychosomatic Research*.

[21] Olejnik S., Algina J. (2003). Generalized eta and omega squared statistics: measures of effect size for some common research designs. *Psychological Methods*.

[22] Cohen J. (1988). *Statistical Power Analysis for the Behavioral Sciences*.

[23] Doron G., Kyrios M. (2005). Obsessive compulsive disorder: a review of possible specific internal representations within a broader cognitive theory. *Clinical Psychology Review*.

[24] Nedelisky A., Steele M. (2009). Attachment to people and to objects in obsessive-compulsive disorder: an exploratory comparison of hoarders and non-hoarders. *Attachment and Human Development*.

[25] Saxena S., Maidment K. M., Vapnik T. (2002). Obsessive-compulsive hoarding: symptom severity and response to multimodal treatment. *Journal of Clinical Psychiatry*.

[26] Abramowitz J. S., Fabricant L. E., Taylor S., Deacon B. J., McKay D., Storch E. A. (2014). The relevance of analogue studies for understanding obsessions and compulsions. *Clinical Psychology Review*.

[27] Lumley M. A. (2000). Alexithymia and negative emotional conditions. *Journal of Psychosomatic Research*.

[28] Lane R. D., Quinlan D. M., Schwartz G. E., Walker P. A., Zeitlin S. B. (1990). The levels of emotional awareness scale: a cognitive-developmental measure of emotion. *Journal of Personality Assessment*.

[29] Waller E., Scheidt C. E. (2004). Somatoform disorders as disorders of affect regulation: a study comparing the TAS-20 with non-self-report measures of alexithymia. *Journal of Psychosomatic Research*.

